# Artificial Intelligence for Automatic Pain Assessment: Research Methods and Perspectives

**DOI:** 10.1155/2023/6018736

**Published:** 2023-06-28

**Authors:** Marco Cascella, Daniela Schiavo, Arturo Cuomo, Alessandro Ottaiano, Francesco Perri, Renato Patrone, Sara Migliarelli, Elena Giovanna Bignami, Alessandro Vittori, Francesco Cutugno

**Affiliations:** ^1^Division of Anesthesia and Pain Medicine, Istituto Nazionale Tumori IRCCS Fondazione G. Pascale, Naples 80131, Italy; ^2^SSD-Innovative Therapies for Abdominal Metastases, Istituto Nazionale Tumori di Napoli IRCCS “G. Pascale”, Via M. Semmola, Naples 80131, Italy; ^3^Head and Neck Oncology Unit, Istituto Nazionale Tumori IRCCS-Fondazione “G. Pascale”, Naples 80131, Italy; ^4^Dieti Department, University of Naples, Naples, Italy; ^5^Division of Hepatobiliary Surgical Oncology, Istituto Nazionale Tumori IRCCS, Fondazione Pascale-IRCCS di Napoli, Naples, Italy; ^6^Department of Pharmacology, Faculty of Medicine and Psychology, University Sapienza of Rome, Rome, Italy; ^7^Anesthesiology, Critical Care and Pain Medicine Division, Department of Medicine and Surgery, University of Parma, Parma, Italy; ^8^Department of Anesthesia and Critical Care, ARCO ROMA, Ospedale Pediatrico Bambino Gesù IRCCS, Rome 00165, Italy; ^9^Department of Electrical Engineering and Information Technologies, University of Naples “Federico II”, Naples 80100, Italy

## Abstract

Although proper pain evaluation is mandatory for establishing the appropriate therapy, self-reported pain level assessment has several limitations. Data-driven artificial intelligence (AI) methods can be employed for research on automatic pain assessment (APA). The goal is the development of objective, standardized, and generalizable instruments useful for pain assessment in different clinical contexts. The purpose of this article is to discuss the state of the art of research and perspectives on APA applications in both research and clinical scenarios. Principles of AI functioning will be addressed. For narrative purposes, AI-based methods are grouped into behavioral-based approaches and neurophysiology-based pain detection methods. Since pain is generally accompanied by spontaneous facial behaviors, several approaches for APA are based on image classification and feature extraction. Language features through natural language strategies, body postures, and respiratory-derived elements are other investigated behavioral-based approaches. Neurophysiology-based pain detection is obtained through electroencephalography, electromyography, electrodermal activity, and other biosignals. Recent approaches involve multimode strategies by combining behaviors with neurophysiological findings. Concerning methods, early studies were conducted by machine learning algorithms such as support vector machine, decision tree, and random forest classifiers. More recently, artificial neural networks such as convolutional and recurrent neural network algorithms are implemented, even in combination. Collaboration programs involving clinicians and computer scientists must be aimed at structuring and processing robust datasets that can be used in various settings, from acute to different chronic pain conditions. Finally, it is crucial to apply the concepts of explainability and ethics when examining AI applications for pain research and management.

## 1. Introduction

Recognizing pain correctly is mandatory to establish the appropriate therapy [1, 2]. Nevertheless, accurate pain evaluation can be a challenging task [3, 4]. Although the expressive subjectivity of the symptom represents the main obstacle, the problems to be addressed are manifold. In some clinical settings, such as cancer pain [5], it is difficult to distinguish the different components of pain [6]. Emotional factors, lifestyle, behavioral components, and personal capacity to face pain are just some of the many elements that may complicate pain assessment [7]. Almost insurmountable problems in pain assessment concern specific categories of patients, such as children with cognitive disabilities [8] and patients of all ages with communication difficulties such as people with dementia [9] or nonverbal, intubated patients [10].

Artificial intelligence (AI) encompasses a wide range of symbolic and statistical approaches to learning and reasoning, emulating several aspects of human brain functioning. There are different classifications based on the processes and characteristics that lead machines to mimic humans in terms of versatility and performance. Machine learning (ML), computer vision (CV), fuzzy logic (FL), and natural language processing (NLP) are subsets of AI. ML is a type of AI that allows systems to learn and improve from experience without being explicitly programmed. It involves training a computer model on a dataset, allowing it to make predictions or decisions without being explicitly programmed to perform the task. Deep learning (DL) is a subfield of AI that is inspired by the structure and function of the brain's neural networks. It involves training artificial neural networks (ANNs), which are made up of layers of interconnected nodes or “neurons,” on large sets of data. These networks are able to automatically learn and extract features from the data. The advent of DL marked a significant turning point in the field of AI, fundamentally changing the way AI systems are developed and applied. Processes of CV, FL, and NLP involve the development of ANNs which, in their complexity, are part of DL [11].

**Figure 1:**
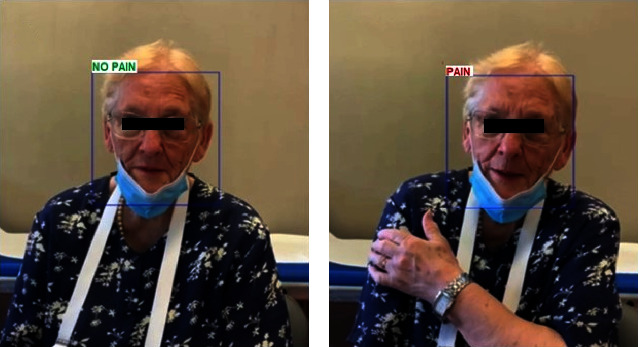
Automatic pain assessment in a cancer patient. “Pain” and “no-pain” states. Pretrained system based on a combination of computer vision and natural language processing methods. In the two selected frames, the system recognizes when the patient passes from a state of absence of pain (a) to a state of pain when she touches her right shoulder (b). The right shoulder is the site of a secondary bone lesion for breast cancer. Patient consent was acquired for the study (clinicaltrials.gov identifier: NCT04726228) and scientific divulgation.

Starting from complex datasets, AI systems can develop predictive modeling tasks also useful for pain research [12]. In particular, data-driven AI models can be adopted to bypass the limitations of subjective pain evaluation. The aim is the development of reliable pain assessment methods based on objective, standardized, and generalizable elements. Overall, these methods are indicated as automatic pain assessment (APA) [13–15] (Figure 1). Despite this ambitious goal seeming to be a chimera to clinicians, research is making progress at an incredible speed. Several research groups worldwide are engaged in the field of APA research, but the lack of knowledge can represent an obstacle to the potential translation into clinical practice. These limitations contribute to the AI chasm phenomenon, namely, the gap between the development of an AI algorithm and its application [16, 17].

The purpose of this article is to review the key research and perspectives on the subject. Principles of AI functioning will be addressed to offer more details to less experienced readers.

In this paper, the AI-based methods are grouped into two categories: (1) behavioral-based approaches including facial expressions, linguistic analyses, and nonverbal physical indicators of pain such as body movements and (2) neurophysiology-based pain detection methods. Although this division is useful for narrative purposes, many approaches involve multimode strategies by combining behaviors with neurophysiological techniques. For example, electromyography (EMG) can be used for developing predictive models using facial expressions. The text will explore both experimental and clinical pain scenarios.

## 2. Behavior-Based Approaches

Affective computing, also known as artificial emotional intelligence or emotion AI, refers to the field of computing that deals with emotions and their influence on human behavior. It encompasses a broad range of topics and uses, one of which is the assessment and representation of affective phenomena such as pain [18]. Pain and other affective processes have observable markers, including facial expressions, language features, body postures, and respiratory-derived elements. These behaviors can be recorded and analyzed using technology.

### 2.1. Facial Expressions

Since pain is generally accompanied by spontaneous facial behaviors, facial expressions can be a useful method for pain evaluation. Notably, it was demonstrated that facial expressions of pain show consistency across ages, genders, cognitive states (e.g., noncommunicative patients), and different types of pain and may correlate with self-report of pain [19]. On these bases, different attempts were conducted by using simple facial images or video recordings. The facial action coding system (FACS) is a manual method for describing and analyzing observable facial movements. It breaks down facial movements into a set of basic units (*n* = 44) called action units (AUs) which correspond to the activation of a specific muscle or group of muscles and can be identified and scored independently from other AUs. The FACS manual provides guidelines for scoring these AUs, using a set of photographs and illustrations as aids [20]. This system is widely used in fields such as psychology, sociology, and communication studies, as well as in the development of facial animation software and the evaluation of facial paralysis. FACS has been used to study the facial expressions of pain in a variety of populations, including healthy individuals, patients with chronic pain conditions, and individuals with neurological or psychiatric disorders [21]. Researchers have used FACS to study the facial expressions of pain in response to different types of pain stimuli, such as thermal, electrical, and pressure stimuli, as well as in response to different types of pain medication [22].

These approaches were burdened with multiple biases such as identifications of typical expressions during analysis (e.g., smile) and differences between cohorts of populations [23]. Moreover, FACS is carried out by human observers who need to go through specialized training to make assessments that can be trusted in scientific research. Undoubtedly, the advancement of AI techniques has changed the landscape stimulating the development of strategies for computer-mediated automatic detection of pain-related behaviors [24].

Several approaches for AI-based image processing have been implemented [25]. It is important to note that the choice of model will depend on the specific task and dataset and that different models may perform better for different tasks.

Image classification and object recognition tasks are usually performed by using convolutional neural networks (CNNs). In brief, CNNs are a type of feedforward ANN where, unlike recurrent neural networks (RNNs), connections between nodes do not form loops. They are high-performance networks that recall the functioning of the retina, mapping one input to one output. In addition to vision tasks (i.e., computer vision), speech recognition is another application of CNNs. Schematically, CNNs are formed by the first layer (convolutional layer) used to detect features, a nonlinearity layer (introduction of nonlinearity into the system), and a series of pooling layers (parameters downsampling) until the fully connected layer (flattening). Each node in the output layer connects directly to a node in the previous layer. In the final neuronal layer, the classification is carried out based on the characteristics extracted through the previous layers and the different filters applied [26]. The CNN architecture may vary, but visual geometry group (VGG) 16 architecture is a reference model for building CNNs. It consists of 16 convolutional layers with 3 × 3 and numerous filters. For pain research, VGGFace is often implemented. It is a variant of the VGG16 and VGG19 models that were using a large dataset of face images (VGGFace2), which contains more than 3 million images of faces from more than 9,000 individuals [27]. The VGGFace model can be fine-tuned for a variety of facial recognition tasks, such as face verification, face identification, and emotion recognition. Other CNN architectures include AlexNet, LeNet, ResNet, and GoogLeNet.

Beyond the CNN, many types of ANNs can be used for image-processing tasks. Deep residual networks (ResNets), for example, are an improvement on traditional CNNs that are able to handle deeper architectures, thus allowing to improve performance. Moreover, generative adversarial networks (GANs) are a type of the ANN that are used to generate new images by using two neural networks: one that generates images and another that verifies if the generated images are similar to the real ones. Another neural network architecture is autoencoder. It can be used to compress and reconstruct images. The autoencoder consists of two main parts: an encoder and a decoder. The encoder is responsible for learning a compact representation of the input data (latent representation), while the decoder is responsible for reconstructing the original input data from this compact representation [28]. Finally, U-Nets are a type of CNN model useful for image segmentation tasks.

Different models of ANNs are used for image processing. According to Yu et al. [29], a dual model can better imitate the human brain's visual functioning. On these premises, a dual CNN model was planned to detect pain from facial expressions. The authors implemented a modified residual neural network architecture and achieved an accuracy of 99% on a pretrained dataset (UNBC-McMaster shoulder pain database) and 90% on unseen subject data [30]. In the case of complex visual data (different dimensionality) that need adequate preprocessing, the Siamese model is often used. It consists of two identical ANNs that work in parallel (tandem working) according to a feedforward and back-propagation flow, and result in comparative outputs. Chang et al. [31] adopted a convolutional Siamese network from magnetic resonance imaging (MRI) for the assessment of knee pain.

Ensemble deep learning models (EDLMs) or fusion models are featured by the integration of two or more algorithms. In EDLMs, the models work together to improve the overall performance of the system. This can be done by combining the predictions of multiple models, or by training a higher-level model to make decisions based on the outputs of lower-level models. According to complex approaches, hybrid models were proposed for pain research. For example, Bellantonio [32] demonstrated that a combination of the CNN and recurrent neural network (RNN) improved spatial and temporal pain data from facial videos. Another EDLM CNN-RNN method was proposed by Bargshady et al. [23]. They used the VGGFace dataset for fine-tuning and the UNBC-McMaster Shoulder Pain dataset as a test dataset.

RNNs are neural networks that are designed to process sequential data such as time series data or natural language text, by using feedback connections that allow information to be passed from one step of the sequence to the next. Consequently, vanishing and exploding gradients with difficult learning of long-term dependencies are the main limitations of traditional RNNs. Thanks to the activation of memory layers called “gates,” the recurrent model long short-term memory network (LSTM) “remembers” the past knowledge of the network (input gates) and “forgets” irrelevant data (output gates). In particular, LSTM is a type of the RNN that is able to effectively learn and remember long-term dependencies in sequential data. The gates are responsible for deciding which information to keep and which to discard in the memory cells, allowing the network to selectively remember or forget information from previous time steps.

LSTMs have been applied to various tasks such as natural language processing, speech recognition, and time series forecasting. A bidirectional LSTM (BiLSTM) network comprises two LSTMs, one that processes the input sequence in the forward direction and one that processes the input in the backward direction. The outputs of these two LSTMs are then concatenated and fed as input to the next layer. The bidirectional nature of the network allows it to consider both past and future contexts when making predictions. Traditional and BiLSTM networks are also used in the field of pain research, where they can be used to analyze physiological signals such as facial expressions, body language, speech, and physiological signals such as heart rate and galvanic skin response [33–35].

A hybrid neural network that combines a CNN with an LSTM network has been proposed for pain research. CNNs are good at extracting features from images, while LSTM networks are good at processing sequential data. Recently, Rodriguez et al. [36] exploited the CNN and LSTM for facial expression classification of different publicly available datasets. The study's approach first adopted CNNs to learn facial features from the VGGFace dataset. These features were then linked to an LSTM network to exploit the temporal relationship between video frames. The study compared the performance of using a schema based on canonically normalized appearance versus using the whole image.

### 2.2. Language Analysis

Language analysis includes language feature extraction and classification. In this regard, the verbal taxonomy of pain represents the starting point for this type of research. The pain descriptor system (PDS) is composed of 24 descriptors and 8 subcategories [37]. Based on this classifier, several types of investigation such as survey analysis on pain issues can be conducted [38].

The application of natural language processing (NLP) represents the evolutionary step in the language analysis for APA. NLP is the field of AI aimed at “exploiting rich knowledge resources with the goal of understanding, extraction, and retrieval (of data) from unstructured (written and spoken) texts” [39]. It focuses on the interaction between computers and human language combining computer science, AI, and linguistics. Classification, annotation, and prediction are the three main NLP methodologies. Tasks of NLP include language translation, text summarization, sentiment analysis, and question answering. NLP is commonly used in a wide range of applications such as chatbots, virtual assistants, and language-enabled applications.

The various phases of NLP include the tokenization (text division into tokens corresponding to spaces, words, punctuation, and sentences), the morphological and lexical analysis, the syntactic analysis and the generation of parse trees, the named entity recognition (information extraction), semantic analysis, and speech analysis. NLP has several practical applications in medicine. For example, it can enable computerized clinical decision support systems, improve healthcare management (feedback analysis), and can be used for building tele-triage services (chatbots) and other aims [40, 41].

The field of NLP faces a unique challenge when it comes to the concept of “pain,” as it is a subjective and often ambiguously described phenomenon [42]. It can encompass physical discomfort, emotional suffering, and other biopsychosocial elements, making it difficult to accurately analyze and understand through text-based data sources. Different lexicons were developed for pain investigations. For example, Chaturvedi et al. [43] validated a lexicon of 382 terms useful for selecting suitable pain-related elements from electronic health record databases.

NLP can be used in pain research and clinical scenarios to analyze and extract information (e.g., pain location, intensity, and duration) from text-based data sources such as electronic medical records, clinical notes, and patient-reported outcomes [42]. This can help researchers understand the patient's experience of pain and identify patterns or trends in pain management. Interestingly, Naseri et al. [44] developed a method helpful for automatically identifying and categorizing pain reported by physicians in clinical notes, even when the pain is not recorded through structured data entry. The MetaMap and NegEx algorithms were used for medical terms' extraction.

In clinical contexts, NLP can be used to automatically summarize clinical notes, and for dialogue systems, such as chatbots, that can interact with patients and help them manage their pain. Furthermore, NLP can be used to develop question-answering systems that can provide patients with accurate and up-to-date information about pain management.

Another application of NLP in pain research is sentiment analysis [45]. It combines ML algorithms (e.g., SVM classifier) and NLP processes for classifying whether a block of text is positive, negative, or neutral. Valence, activation (depression), and identification of the arousal component of emotion are usually investigated. This approach can be used for investigating patient-reported outcomes and analyzing the language used in patient surveys or online patient forums to understand patients' emotions and opinions about their pain and treatment.

Several algorithms were improved for NLP applications in pain research. For example, Word2Vec is a two-layer ANN and GloVe is an unsupervised ML algorithm. They work on datasets of representative words, sentences, and phrases in a given language for a given argument (linguistic corpora). A recent systematic review was conducted for evaluating applications of NLP in low back pain and spinal disease [46]. In the final analysis, the authors included 16 articles and collected different rule-based [47] and supervised or unsupervised ML approaches [48].

**Figure 2:**
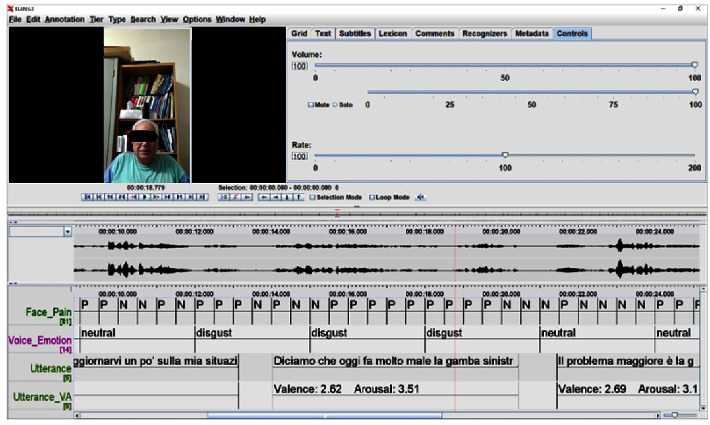
The ELAN tool (version 6.3) is implemented to combine and analyze frame-by-frame facial expressions (pain/no-pain) and language analysis including textual phonetic and prosodic analysis, sentimental analysis (e.g., neutral and disgust), and arousal. Patient consent was acquired for the study (clinicaltrials.gov identifier: NCT04726228) and scientific divulgation.

For pain research, language analysis can be combined with the facial expression analysis. For example, the ELAN tool is open source under the GNU General Public License. It can be used to assemble different behavioral features (Figure 2).

### 2.3. Other Behaviors

In a fascinating article, Walsh et al. [49] investigated links between body posture and pain. They planned a stimulus set and, during the validation stage, highlighted that “head averted,” “gaze downward,” and “forward body lean” are common body postures for pain as performed by actors. On these findings, it was suggested that reduced head motion and altered postures could be used as pain behaviors. For instance, Werner et al. [50] found that head movements and postures tend to be oriented downwards or towards the pain location.

Recently, Cao et al. [51] extracted potential pain-related respiratory features from photoplethysmography (PPG) in postoperative patients included in the UCI iHurtDB pain protocol [52]. They implemented five ML algorithms including ADABoost, XGBoost, random forest, SVM, and KNN classifiers. The accuracies were satisfactory for all five classifiers, and the authors compared their results with those obtained by Thiam and Schwenker [53] who used 65 automatic respiratory features using an appropriate fusion architecture method (Table 1).

## 3. Neurophysiology-Based Pain Detection

Neurophysiology-based pain detection is a method of measuring and assessing pain that relies on the study of the physiological changes that occur in response to pain. The field is dynamic and continuously advancing, with new research uncovering new areas for exploration.

### 3.1. Electroencephalography

Accumulating evidence suggested that chronic pain is associated with structural and functional changes in the brain [54]. Interestingly, electroencephalography (EEG) can be used to track these changes and thus utilized for investigating biomarkers of pain [55]. For example, typical neuronal activities in the sensorimotor cortex, such as an increase of theta and gamma oscillations, can be the expression of distinct pain states [56]. Moreover, it was found that the gamma band is a predominant predictor of acute thermal pain [57] and peak alpha frequency recorded at the bilateral temporal scalp was linked with a verbal pain report during stimulation and at rest [58].

In a recent investigation, Chen et al. [59] proposed a multilayer CNN model for objective EEG-based pain detection. Ten volunteers underwent a series of 15 movement tasks (M) (e.g., jogging on a running machine) and watched a set of 15 short videos (V) related to pain scenes. After data acquisition and preprocessing, the model validation was performed for testing the algorithm's ability to distinguish between “nonpain” and “pain” states. In AI analyses, the overall performance of a model is given by the area under the receiver operating characteristic curve (AUC). Reducing the false positive rate and, at the same time, increasing the true negative rate is finding a trade-off cut point between error rates. An AUC of 0.7 to 0.8 is considered acceptable, 0.8 to 0.9 is excellent, and more than 0.9 is an outstanding result. In their analysis, the AUCs were 0.83 and 0.81, in M and V, respectively.

Other attempts with EEG datasets were conducted. Misra et al. [56] used a support vector machine (SVM) algorithm. It is a typical supervised ML algorithm that receives precataloged data as input for decision-making processes (output). SVM maximizes the margin by minimizing the classification error and expresses a binary classification (e.g., pain/no-pain). Levitt et al. [60] used an SVM for obtaining pain phenotypes from EEG features. The study collected EEG data from 20 individuals suffering from chronic lumbar radiculopathy, 20 healthy individuals, and 17 patients affected by chronic lumbar pain and scheduled for neuromodulation therapy.

K-nearest neighbor (KNN) is another ML classifier. It does not require a training phase and is highly sensitive to noisy samples. When a dataset contains both categorical and numerical attributes, KNN and SVM can be used for developing a decision tree. It seeks the best split to subset the data. More recently, Nezam et al. [61] collected EEG and electromyogram (EMG) signals and used SVM and KNN decision tree models for evaluating different pain levels. The classification accuracies were over 80% for both models.

In another study, Elsayed et al. [62] used a combination of signal processing techniques and ML strategies to analyze brain signals related to pain and categorize them into four levels of pain intensity (no pain, low, moderate, and high). They discovered that the signal processing revealed a direct link between the alpha frequency band power and the level of pain. The classifier developed had an accuracy of 94.83%. These results were supported by other studies, indicating that the normalized alpha power in the central region of the brain may serve as a reliable and quantifiable marker for chronic pain, with potential for clinical use [63].

Overfitting and underfitting are typical ML issues (model fitting errors). Overfitting occurs when the learning of a function adapts very well to the training data but is unable to generalize other information (test set). On the contrary, underfitting occurs when a model performs poorly on the training set. Although decision trees are easy-to-use algorithms, they can be affected by model fitting errors. Random forest (RF) is another ML and can be used for overcoming this problem. It combines the output of multiple decision trees for obtaining a single result (yes/not). In healthy subjects, Vijayakumar et al. [64] trained an RF model to predict pain scores. Tonic thermal stimuli (from a thermal stimulator) were used to mimic pain responses, and EEG data were achieved. The intrasubject and intersubject accuracies were 93% and 89.5%, respectively.

Creating predictive models can be highly valuable in the field of pain medicine, particularly for complex conditions of chronic pain that are challenging to treat. In these clinical scenarios, patients should receive prophylactic treatments. Vuckovic et al. [65] used the ANN, SVM, and linear discriminant analysis (a supervised learning method used to identify the linear combination of features that best separates two or more classes) to recognize spinal cord injured individuals at risk of developing central neuropathic pain. For the three considered models, the accuracy was higher than 85%. Finally, it was recently demonstrated that EEG features can be also used to predict the effects of pain treatments [66].

### 3.2. Electrodermal Activity

The link between pain and autonomic nervous system activity is an interesting field of study. The eccrine sweat glands have the highest density on palmar/plantar surfaces of hands/feet (600 to 700 glands/cm^2^). Since there is a predominant sympathetic innervation, these glands are activated within the fight-or-flight response. Furthermore, they are more responsive to psychological stimuli than to thermal inputs. Thus, their activation can represent a valid means to study objective responses to pain.

**Figure 3:**
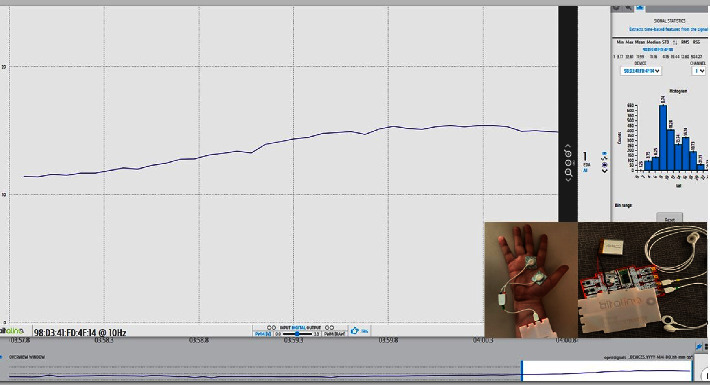
Electrodermal activity recorded through the BITalino® platform (in the box).

Also known as skin conductance, galvanic skin response, and sympathetic skin response, electrodermal activity (EDA) is the continuous variation in the electrical characteristics of the skin, which varies with the moisture level. Concerning the physical functioning basis, a low constant voltage current is passed through a pair of electrodes placed on the surface of the skin. With a constant voltage, it is possible to measure the current which varies directly with the skin conductance (measured in *µ* Siemens). Several wearable noninvasive devices are available. For example, EDA is integrated into Internet-of-Things devices such as the BITalino® multichannel platform. It is an open-source biosignal platform compatible with easy-to-use software such as OpenSignals that can be used for obtaining data from electrocardiography (ECG), EMG, electrodermal activity EDA, and EEG (Figure 3).

Empatica E4 Wristband (Empatica Inc, Boston, MA, USA) is another device for EDA and PPG recording. It also measures heart rate and motion-based activity (accelerometer *x*-, *y*-, and *z*-axes), as well as skin temperature and can mark events through a tag button. This wearable device is primarily used for research in fields such as psychology, neuroscience, and physiology. It is also used for monitoring stress and emotional states, as well as for tracking sleep patterns and physical activity in chronic pain rehabilitation [67], and for monitoring opioid use in patients with pain conditions [68].

Due to these characteristics, EDA would have space in acute pain research or for investigating typical acute pain phenomena in the context of cancer pain, such as breakthrough cancer pain. Different EDA-based studies for APA were conducted. For example, Susam et al. [69] distinguished pain levels in children who underwent surgery (laparoscopic appendectomy). Moreover, Gruss et al. [70] created a database of EDA and other biopotentials (EMG and ECG) collected on healthy participants (*n* = 85) subjected to painful heat stimuli. They implemented SVM and obtained an accuracy of approximately 90% when the pain tolerance threshold was compared to the baseline.

The EDA approach has several limitations and must be well placed in the context of the multiparametric analysis. Several clinical experiences have shown that there is notable variability in EDA measurement. This method also appears to have sensitivity but poor specificity in pain assessment. Variations in tonic signals and latency in phasic activation (acute pain) are recognized challenges to be addressed. Consequently, the data analysis must include an accurate timescale decomposition to extract salient features from the original signal (preprocessing stage). Despite limitations, the technique has ample room for improvement and several approaches have been proposed to improve its accuracy. For example, Hossain et al. [71] proposed an ad hoc algorithm to solve the problem of noise and motion artifacts.

### 3.3. Other Neurophysiological Methods

Heart rate variability (HRV) is a physiological measure that reflects the variation in time between successive heartbeats. It is considered an important indicator of the body's ability to regulate itself and maintain homeostasis. HRV is often used as an indirect measure of the activity of the autonomic nervous system (ANS). Moreover, HRV is also an important marker of emotional processing. Emotions such as stress and anxiety can lead to changes in HRV, which can be measured and used to assess emotional states. HRV can be measured through various techniques such as electrocardiography (ECG) or PPG.

PPG is a noninvasive optical method that measures fluctuations in blood volume by using a light source and a corresponding photodetector. The light source illuminates a part of the body's surface, including the skin and blood vessels, and the photodetector detects the variations in light (either reflected or transmitted, depending on the PPG sensor design), which are modulated by the pulsatile blood flow. This blood flow is largely determined by factors such as the heartbeat, the rigidity of blood vessels, and the respiratory rate [72]. In recent years, advancements have been made in the field of PPG, with researchers developing automatic classifiers to detect PPG pulses. These classifiers are designed to recognize the unique morphological characteristics of the PPG signals, which are indicative of the pulse waveform. By utilizing these classifiers, it should be effectively ameliorated the selection of PPG features that are suitable for further processing and analysis. This is an important step in obtaining accurate and reliable information from PPG signals and ultimately advancing our understanding of various physiological processes, including pain-related phenomena [73].

Research indicated that a higher level of HRV at rest reflects a state of highly adaptive emotional responses, while low HRV is linked to various health issues such as cardiovascular disease, mood disorders, and increased risk of disease [74]. Concerning HRV applications for pain management, despite the expectative, a recent systematic review found limited evidence of its efficacy for chronic pain assessment [75].

Other investigated hemodynamic parameters are the systolic and diastolic pressure values. In a recent systematic review, Moscato et al. [76] found that among various physiological signals, blood pressure and parameters obtained from ECG are most widely investigated. In particular, the low-frequency/high-frequency (LF/HF) ratio, which is derived from ECG, has received significant attention. Studies have shown that there is a positive relationship between pain and several physiological signals, including HRV, LF/HF ratio, and systolic blood pressure.

Functional MRI and positron emission tomography (PET) are other neurophysiological techniques that can be used to detect pain-related changes in the brain activity. These methods are implemented for research aims [77, 78].

Surface EMG (sEMG) is a promising technology in automatic pattern recognition. It uses noninvasive electrodes placed on the skin to measure the electrical activity of superficial muscles. sEMG has the advantage of detecting subtle facial muscle activity that may not be visible to the naked eye. However, a recent evidence-based analysis found only one study that used sEMG to objectively detect facial pain expressions [79]. As a result, the proposed correlation between muscle tension and pain intensity requires further research [80].

The advantages and disadvantages of behavior-based approaches and neurophysiology-based modalities are shown in Table 2.

## 4. Research Perspectives and Issues

Research in this field must address several issues. A key aspect is multimodal data collection. It must address the different settings of acute (e.g., postoperative pain) and chronic pain (benign and cancer-related) and must be performed in clinical scenarios that require special attention such as patients with communication difficulties [6, 8]. The quality of the data, suggested by the “Incredible Five V's” that include variety, velocity, volume, veracity, and value [81], must presuppose the dynamism of their acquisition. In other words, big data must be well structured, but this is not enough as there is a need for continuous updating. The performance of AI systems depends on it. Furthermore, due to the complexity of cancer pain phenomena [82], it is important to capture physiological signals in real-world settings, even using wearable devices [13].

In the context of multimodal datasets, data from multiple measures could be included. Pupillometry, for example, can offer interesting study perspectives, and research on its applications for pain assessment is currently scarce [83]. Data from body temperature, hormonal analyses, genetics (changes as a result of chronic pain), and brain scan-derived measures such as near-infrared spectroscopy, cerebral blood flow velocity, positron emission tomography, and single-photon emission computer tomography could be used for implementing multiparametric datasets [84]. Furthermore, in chronic osteoarticular pain, MRI findings were used to discriminate painful from nonpainful knees [31]. It suggests that imaging data can be valuable elements for structuring a reference dataset.

Several datasets are publicly available for AI-based pain research (Table 3). They include the VGGFace2 [27], the BioVid Heat Pain Database (BioVid) [85], and the UNBC-McMaster Shoulder Pain dataset acquired from individuals suffering from shoulder pain [86], as well as the BP4D-Spontaneous Database (BP4D) [87], the Multimodal Intensity Pain (MIntPAIN) [89], the EmoPain [90], and the SenseEmotion Database obtained through heat stimulation in healthy volunteers [88]. The research community can also use classic datasets designed for automatic facial image analysis such as the Cohn Kanade + facial expression database [91]. All these databases can be used for model testing and evaluation.

A serious gap to be faced is that most of the proposed methods were evaluated on stimulated pain collected from healthy participants. Nevertheless, multimodal datasets were collected from patients suffering from postoperative pain [52] and chronic musculoskeletal pain [86, 90]. Despite these acquisitions, there is a need for the development of pain datasets in key settings, such as cancer pain [92], and primary chronic pain conditions [93]. These datasets should also collect data from children [94], elderly individuals, or individuals with disabilities [95] for tailoring interventions to specific populations.

The analysis of large datasets with diverse compositions, which can include a combination of numerical data, images, and patient-generated descriptions of symptoms, poses significant challenges in the field of APA. The ultimate goal of this analysis is to extract valuable information from the data that can be transformed into knowledge about pain [96]. As a consequence, the choice and proper use of the AI system have a key role in improving results.

When approaching the study of bio parameters, a number of factors must be taken into account. Cancer pain research and investigation on APA should prioritize examining the underlying pathophysiological mechanisms and evaluating the effectiveness of study models for different types of pain [97]. Cancer-induced neuropathic pain, for instance, has distinct pathophysiology, and this issue should be carefully addressed during the collection and analysis of physiological signals [98]. Moreover, distinct autonomic dysfunctions can be produced by anticancer therapy [99, 100], and a bias in the analysis.

There are several factors that can impact the dependability of physiological parameters. For instance, variables such as physical activity, age, sex, and health status can affect the quality of PPG signals and PPG waveform parameters. Of these variables, physical activity has a negative impact on the quality of the PPG output [101].

Finally, research must address explainability and ethics issues. They are critical components of responsible AI, which is the practice of developing and using AI in a way that is transparent, accountable, and aligned with human values. Explainability in AI refers to the ability of a model to provide a clear and understandable explanation of its decision-making process [102]. This is important because it allows stakeholders, such as users, regulators, and developers, to understand how the model arrived at its conclusions and identify any potential biases or errors [103]. Ethics in AI refers to the moral principles and guidelines that govern the development, deployment, and use of AI systems. This includes issues such as privacy, fairness, transparency, accountability, and human autonomy [104]. Ensuring that AI systems are designed and used in an ethical manner is crucial for protecting the rights and welfare of individuals and society as a whole [105]. It is essential to also incorporate the principles of explainability and ethics in the examination of AI applications related to pain research and management.

## 5. Conclusion

Research in the interdisciplinary field of APA can benefit from the utilization of various AI techniques. The field is constantly evolving, and new research is constantly shedding light on new areas for exploration. Although the verbal report is sometimes characterized as the “gold standard” for pain assessment, it remains problematic from a scientific perspective for numerous reasons, including its subjectivity and uncertain underlying metric properties and reliability. While AI can be a great opportunity for developing tools for objective pain evaluation, the pathway development passes towards the creation and analysis of big data and metadata (data about data). The strengthening of collaboration programs must provide for the structuring of datasets that can be used in different settings, from acute pain to the different forms of chronic pain. Finally, the principles of explainability and ethics must be considered in the study and use of AI applications in pain research and management.

## Figures and Tables

**Table 1:** Selected methods for pain behavior research.

Behavior	Method	Notes	Ref.
Facial expressions	Dual CNN	Development of an image classification model	[29]
	Siamese network	MRI for the assessment of knee pain	[31]
	CNNs and LTSM	Image analyses	[36]
	CNN and RNN	Spatiotemporal pain recognition	[32]
	CNN and RNN	VGGFace dataset for fine-tuning and the UNBC-McMaster shoulder pain dataset for testing	[23]

Language analysis	MetaMap and NegEx algorithms	Automatic extraction and classification of physician-reported pain from clinical notes in cancer patients	[44]
	Different ML and ruled-based algorithms	A systematic review on NLP for LBP	[46]
Body posture	16 actors posed in various body postures to depict pain, and 20 observers selected the most effective images. After validation, a set of 144 images was established	“Head averted,” “gaze downward,” and “forward body lean” are common body postures for pain	[49]

Respiratory features	ADABoost, XGBoost, RF, SVM, and KNN	Features from PPG in postoperative patients	[52]
	Fusion architectures	65 automatic respiratory features	[53]

CNN: convolutional neural network; MRI: magnetic resonance imaging; LSTM: long short-term memory network; RNN: recurrent neural network; ML: machine learning; NLP: natural language processing; LBP: low back pain; RF: random forest; SVM: support vector machine; KNN: k-nearest neighbors; PPG: photoplethysmography.

**Table 2:** Advantages and disadvantages of behavioral and neurophysiological approaches.

	Advantages	Disadvantages
*Behavioral methods*		
Facial expressions	Consistency across ages, genders, cognitive states (e.g., noncommunicative patients), and different types of pain. They may correlate with self-report of pain	Complex processing
Language analysis	Useful for sentiment analysis and more suitable for text extraction (e.g., from electronic medical records)	High-complexity processing requiring proper pain taxonomy should be combined with other methods

*Neurophysiology-based*		
Electroencephalography	Correlation with structural and functional changes in the brain	Better suited for experimental settings rather than clinical use
Electrodermal activity	Easy to use	Good sensitivity but poor specificity
Heart rate variability	Easy to use	Poor reliability

**Table 3:** Selected datasets for pain research.

Dataset	*n*	Setting	Included features	Ref.
VGGFace2	9000	Healthy subjects	Facial expressions	[37]
BioVid Heat Pain Database (BioVid)	90	Healthy subjects	Videos and physiological data	[85]
UNBC-McMaster Shoulder Pain Dataset	129	Shoulder pain	Facial expressions, pain frame-by-frame scores, and observer measures	[86]
BP4D-Spontaneous Database	41	Healthy subjects	Facial expressions and head poses	[87]
SenseEmotion Database	45	Healthy subjects	Multimodal sensory data^*∗*^	[88]
Multimodal iIntensity Pain (MIntPAIN)	20	Healthy subjects	Facial expressions	[89]
EmoPain		Chronic pain	Multimodal dataset ^	[90]

°Electrocardiography, photoplethysmography, electrodermal activity, facial expressions, and body postures. ^*∗*^Biopotentials, camera images of the facial region, and audio signals. ^Face videos, audio signals, and electromyographic signals from back muscles.

## Data Availability

The datasets used and/or analyzed during the current study are available from Marco Cascella on reasonable request.
